# 

**Published:** 1912-09-21

**Authors:** 


### Buildings Contemplated and in Progress.

Birmingham.—The Corporation have approved an expenditure of over £29,000 for extending the Yardley Road Sanatorium.

Bourne.—The Rural District Council has approved plans for an isolation hospital, but referred back two consumptive pavilions. The scheme is estimated to cost £4,850.

Bury St. Edmunds.—Tenders are invited for extending the Guardians' Infirmary. Quantities may be had, on deposit of £5, from the architect, Mr. S. Naish, M.S.A., 30 Abbeygate Street, Bury St. Edmunds.

Cairo.—The Chief of the Administrative Service invites tenders for three new pavilions at Khanka Mental Hospital (Qaliubia).

Cheshire.—The Cheshire County Council has resolved that sanatorium accommodation should be provided for the county, in combination with the boroughs of Birkenhead, Chester, Stockport (including Lancashire portion), Wallasey, and Stoke-on-Trent, and that the county medical officer of health confer with the borough officers to frame a joint scheme for further consideration, and also report on the utilisation of existing infectious hospital or sanatoria buildings. It is suggested that dispensaries be established in the boroughs of Birkenhead, Chester, Stockport, Wallasey, and Stoke-on-Trent, and that a sanatorium be erected in the locality of Delamere Forest, with 250 beds, at a cost of about £37,500.

Dorchester.—Messrs. Hine and Pegg are architects to provide accommodation at Dorchester Mental Hospital for fifty more female beds. The plans have been approved by the Commissioners in Lunacy, and the cost is estimated to be £5,250.

Glasgow.—The Glasgow Corporation Health Committee has resolved to establish a consumptive colony at an initial expenditure of £60,000 for 210 patients, but the plans permit of further accommodation to a total of 600.

Glasgow.—Rapid progress is being made with the new buildings at Yorkhill for the Royal Glasgow Sick Children's Hospital. Dr. Mackintosh, M.V.O., is advisory medical expert, collaborating with the architect, Dr. J. J. Burnet, A.R.S.A.

Gloucester.—Mr. Walter B. Wood, A.R.I.B.A., of Gloucester, is architect for a proposed new infirmary in Great Western Road. Quantities have been prepared by Messrs. Vale and Kingsford, also of Gloucester, and may be had on deposit of £5.

Grimsby.—The Grimsby Guardians have given instructions for the preparation of a design extending the workhouse buildings and including a new infirmary.

Haslingden.—The Guardians are seeking sanction from the Local Government Board to a loan of £4,000 for a new infirmary and boiler house.

Hinckley.—Plans for a proposed isolation hospital, to cost about £5,000, have passed committee, and are being forwarded to the Local Government Board for approval.

Horsham.—Messrs. Wheeler and Godman, of Horsham, are architects for alterations at the Guardians' Infirmary, and particulars for tenders may be had from them on application.

Keighley.—The Keighley and Bingley Joint Board has approved plans for a cubicle isolation pavilion to cost about £2,500.

London.—The Shoreditch Guardians propose to rearrange wards at the workhouse according to plans prepared by Mr. F. J. Smith, F.R.I.B.A., Parliament Mansions, Victoria Street, S.W. Tenders are invited.

Menston.—The Wharfedale Union Joint Isolation Hospital Committee have decided to extend the hospital accommodation by the addition of thirty-two beds and to provide a board-room and extra nurse accommodation. The cost is estimated at £3,000.

Pontladies.—The Llandilo Guardians propose to erect an infirmary, to cost £4,000, on a site at Pontladies.

Surrey.—The Surrey County Council has authorised an expenditure of £5,000 for the provision, equipment, and staff costs of four chief dispensaries and a bacteriological laboratory, together with temporary hospital and sanatorium accommodation, pending permanent arrangements.

Willesden.—The Willesden Urban Council has formulated a scheme for approval by the Local Government Board for a sanatorium and dispensaries to serve the district.

Wokingham.—The Wokingham Guardians have requested the medical officer to report on the proposal to provide isolation hospital accommodation.

Wonthaggi.—It is reported that the Victorian Government is to erect a hospital at Wonthaggi to cost £8,000.

Yorkshire.—A tender of £1,185 has been accepted for minor additions to the North Riding Mental Hospital and a pavilion for tuberculosis cases.

Yorkshire.—The accommodation at the Wharfedale Isolation Hospital, situate at Menston, being inadequate to the requirements, the committee invited local architects to submit schemes for new diphtheria and convalescent pavilions and increasing the administrative quarters. Four schemes were submitted, and that prepared by Messrs. Marshall and Son has been selected. The cost of the scheme is close on £4,000.

### BUREAU OF INFORMATION.

### Rules for Correspondents.

1. Every letter, on whatever subject, **must be accompanied by the coupon** to be cut from the back cover (inside page) of The Hospital, current issue, and must contain the name and address of the correspondent with pseudonym for publication if desired.

2. Replies by post cannot be given, save under exceptional circumstances at the Editor's discretion.

3. Letters from Approved Homes in reply to special needs published in the Bureau should state terms and full particulars, and be sent prepaid under cover to the Editor of the Bureau with coupon and name enclosed for identification.

4. Proprietors of Homes which have not yet been entered on the List of Approved Homes, but have spare accommodation likely to suit special needs, are invited to write for an application form for registration. The fee for registration, which includes two announcements of the Home in the Bureau, is 5s.

5. Special needs of every description relating to those who are sick or in difficulties are made known in these columns **free of charge.**

6. All communications to be addressed to The Editor of The Hospital, 28 Southampton Street, Strand, London, W.C., and marked "Bureau of Information."

### Needs and Helpers.

**Home Offered.**—The best of thanks are due to the Hon. Secretary of the Southport Homœopathic Cottage Hospital for kind offer in connection with F. M. D.'s little boy, recovering from nephritis.

**Convalescent Home Wanted.**—After so serious an operation as amputation of the leg owing to diabetic gangrene you must necessarily be in a weak state, and we are not surprised that you are still out of employment. We recommend you to apply for admission to the Merchant Taylors' Company's Convalescent Home for Men at Bognor. Write to the Clerk, The Hall, Threadneedle Street, London, E.C. It is quite free, and surgical cases such as yours are preferred. If you like to send us particulars of your circumstances and a reference we may be able to help you in the other matter.—A. Burgess.

**Home for Female Epileptic.**—We have much sympathy with your desire to make a comfortable arrangement for your helpless wife while you are away on foreign service. What are you paying for her now, and could you give 15s. a week? We think, if so, we could place her where she would have very kind care.—L. Baker.

**Aid in First Confinement.**—We are greatly indebted to Sister E. M. for her generous help in connection with "Home Wanted."

**Patients with Small Means.**—You ask if there are any homes for persons who can only afford to pay from 10s. to 18s. a week, and you state that within six months you have had seven such cases asking to be received. There are several homes on a philanthropic basis for patients who can only pay very low terms, but alack! they are always full. We are quite sure that no home for invalids could be run on these terms and pay a dividend on the capital invested; it is very doubtful if it could pay running expenses. But experience has shown us that numerous nursing homes are generously willing to take in a case of this kind to fill a vacant bed, and we know of more than one home where a ward has been arranged at very low terms for patients who require kind care rather than acute nursing. If you will send on to the Bureau any cases you are not able to deal with yourself, we shall be very pleased to make an announcement of their needs. We want all our Approved Homes to co-operate with us to this extent by letting patients who are not able to afford their usual terms know of the opportunity we offer them of finding accommodation suited to their purse. Many thanks for your own kind offer to Sister E. M.—Chronometer.

### Approved Homes.

Note.—*No Home is added to the List without direct medical and other testimony to its good management.*

**Lowestoft.**— The Alexandra Home, 407-409 London Road, South Lowestoft. Proprietors: Nurses E. and M. Manwaring. Medical, surgical, maternity, chronic, and convalescent cases. Bracing air, near sea. Good operating theatre. Patients visited by their own doctors.

**For Nerve Cases at Brixton.**—3 Lorn Road, S.W. Nurse McCoy receives slight mental and nerve cases. Massage, electrical and Nauheim treatment. Experienced and successful with nervous patients. Nice garden and every comfort.

**Blackpool and St. Anne's.** — Nurses' and Nursing Homes: 112 Hornby Road, Blackpool; 17 Fairhaven Road, St. Anne's-on-the-Sea. Superintendents: Miss Mary M. Strange and Sister Grace Strange. Medical, surgical, slight mental, maternity, chronic, tuberculosis, and convalescent cases received. Near sea. Garden and greenhouse.

### Training and Employment.

**Hotel Nursing Abroad.**—The best way to procure a post is to apply direct to the principal hotels on the Riviera and in other places resorted to by invalids in the winter. The letter should be distinctly written and contain two or three typed testimonials. As a rule the salary is very small, and sometimes it is nil, board and residence being considered sufficient equivalent in these favoured spots during the season, especially as the work is intermittent. All these engagements are made early in the season, and we fear you are already too late.—Nurse Ethel.

**Dispensing for Women.**—The first step is to pass a qualifying examination if you have not already done so. The Junior Oxford or Cambridge Local suffices, and a list is obtainable from the Secretary of the Pharmaceutical Society, 17 Bloomsbury Square, London. The qualification of "registered chemist" is only granted after a studentship or apprenticeship of three years, leading on to the Minor Examination of the Pharmaceutical Society. The names of lady chemists who take pupils can be obtained from the Secretary as above. Apprenticeship fees vary from £50 to £100, and the course at a school of pharmacy costs from £15 to £30 in addition.—Radium.

**Slight Physical Defect.**—After careful consultation with the head of a large training school, we are disposed to think you would make a better career in a profession where you would not be constantly reminded of what is such a very trifling matter in all but a nursing point of view. There can be little doubt it would draw upon you constant unfavourable criticism, both from doctors and fellow-nurses. Why not make application to be trained instead as a Hospital Almoner (see The Hospital Bureau, August 24) or as Health Visitor? This kind of work demands similar qualities to that of a nurse, and we should judge you were admirably fitted by education and temperament to fill any post requiring tact, sympathy, and patience. It is wiser to undertake work in which you can excel rather than attempt to get training in a small hospital. Shall be glad to hear again if we can be of further use.—Dedication.

### Letter-Box.

The Secretary Sister, Clewer.—Best thanks for the information relating to the works of the Community.

**A leaflet containing particulars of the Bureau will be forwarded free on application.**

### Gifts and Bequests.

Mr. Graham Vivian, of Clyne Castle, Blackpyll, Glamorgan, has left £10,000 to Swansea Hospital.

Mr. William Ansell Todd, of Portishead, Somerset, retired clothing manufacturer, has bequeathed £250 each to the Bristol Royal Infirmary, the Bristol General Hospital, the Bristol Eye Hospital, and the Bristol Hospital for Sick Children and Women.

Mr. Frank Newton Hobson, of Nottingham, managing director of Messrs. Swift and Wass, Limited, curtain machine builders, who started life as an errand boy, and who left estate valued at £110,203 gross, has bequeathed £500 each to the Nottingham General Hospital and the Nottingham Children's Hospital, £200 to the Nottingham Consumption Sanatorium, and £100 each to the Nottingham General Dispensary and the Samaritan Hospital for Women, Nottingham.

